# Association Between Long Term Exposure to PM_2.5_ and Its Components on Severe Obesity in Chinese Children and Adolescents: A National Study in China

**DOI:** 10.3390/children11121536

**Published:** 2024-12-18

**Authors:** Tongjun Guo, Tianjiao Chen, Li Chen, Jieyu Liu, Xinli Song, Yi Zhang, Ruolin Wang, Jianuo Jiang, Yang Qin, Ziqi Dong, Dengcheng Zhang, Zhiying Song, Wen Yuan, Yanhui Dong, Yi Song, Jun Ma

**Affiliations:** 1 National Health Commission Key Laboratory of Reproductive Health, Institute of Child and Adolescent Health, School of Public Health, Peking University, Beijing 100191, Chinadzq1020@pku.edu.cn (Z.D.); songyi@bjmu.edu.cn (Y.S.);; 2 UNESCO Chair on Global Health and Education of Peking University, Beijing 100191, China

**Keywords:** children, PM_2.5_, severe obesity, components, adolescents

## Abstract

Background: The aim of this study was to explore the association between long-term exposure to particulate matter with an aerodynamic diameter <2.5 μm (PM_2.5_) and its components and severe obesity in children and adolescents. Methods: Data for children and adolescents aged 9–18 in this cross-sectional study were obtained from the 2019 Chinese National Survey on Students’ Constitution and Health (CNSSCH). Data for PM_2.5_ and its components were obtained from the Tracking Air Pollution in China (TAP) dataset and matched with information on these children. Logistic regression models were used to assess the risk of severe obesity associated with long-term exposure to PM_2.5_ and its components. Results: A total of 160,205 children were included in the analysis. Long-term exposure to PM_2.5_ may increase the odds of severe childhood obesity, with this effect being more pronounced in girls. Among boys, the component with the most significant impact on severe obesity was organic matter (OM). The impact of PM_2.5_ and its components on severe obesity was greater in children from low economic and low parental education level households. Children with unhealthy lifestyle habits have higher odds of severe obesity due to long-term exposure to PM_2.5_ and its components. Conclusions: The findings of this research support the development of strategies aimed at addressing severe obesity in children, suggesting that adopting healthy lifestyle practices could mitigate the odds of severe obesity due to PM_2.5_ and its components. There is a need for an increased focus on children in economically underdeveloped areas and those with unhealthy lifestyle habits, particularly those in rural areas and those who do not engage in adequate physical activity or get enough sleep.

## 1. Introduction

With the continuous increase in the global obese population, obesity has become a serious public health issue. The phenomenon of childhood obesity in our country is becoming increasingly prominent, and its negative impact on health cannot be ignored [1,2]. Childhood obesity not only significantly increases the risk of developing chronic diseases such as cardiovascular disease, diabetes, and hypertension but may also have long-term effects on children’s mental health [3,4,5]. For example, childhood obesity is closely associated with issues such as anxiety, depression, and low self-esteem [6]. This not only manifests in physiological aspects but also impacts a child’s social adaptation ability and academic performance [7]. Children with obesity are more susceptible to bullying and stigmatization by their peers, which can lead to social isolation, low self-confidence, and negative impacts on their mental health and academic trajectory [8]. Studies have shown that the risk of related health hazards is higher in severely obese individuals [9]. For instance, when an 18-year-old male whose body mass index (BMI) rises from underweight to very obese, his lifetime risk of developing type 2 diabetes jumps from 7% to 70% [10]; individuals with severe obesity also exhibit a significantly increased risk of cardiovascular disease compared to those with normal weight [11]. These health hazards are also associated with a higher mortality rate [12].

In recent years, epidemiological evidence suggests that exposure to particulate matter with a diameter of less than 2.5 μm (PM_2.5_) may be a potential factor influencing severe childhood obesity [13]. However, a scientific consensus on the relationship between PM_2.5_ exposure and severe childhood obesity has not yet been established, as many research results show inconsistent trends. This inconsistency may stem from significant differences in the chemical composition of PM_2.5_ in different regions, with diverse effects of different components on severe childhood obesity [14]. For instance, certain specific chemical components may exacerbate obesity risk by affecting metabolic pathways or the endocrine system [15]. Studies suggest that PM_2.5_ exposure may affect children’s physical development and dietary habits, thereby influencing their risk of obesity. For example, PM_2.5_ exposure has been linked to increased weight and BMI in children, particularly during early childhood [16]. Furthermore, PM_2.5_ exposure may also affect children’s health behaviors, such as their appetite and food choices [17,18]. It is worth noting that more research is needed to establish a scientific consensus on the relationship between PM_2.5_ exposure and severe childhood obesity.

Currently, no studies have reported an association between PM_2.5_ components and the development of severe obesity, and the underlying mechanisms remain unexplored. Therefore, this study aimed to investigate the association between long-term exposure to PM_2.5_ and its components and severe obesity in children and adolescents. This research will provide targeted reference for the development of effective prevention strategies to help improve children’s health.

## 2. Methods

### 2.1. Study Population

**Figure 1:**
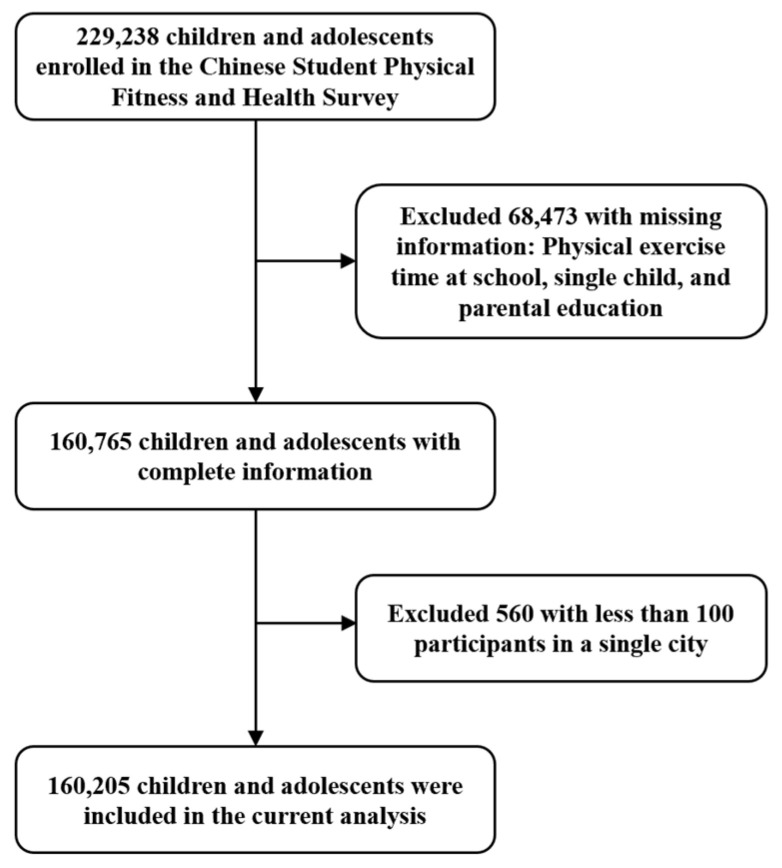
Flow chart of study participants.

This study utilized data from the 2019 Chinese National Survey on Students’ Constitution and Health (CNSSCH), a cross-sectional survey conducted every five years to assess the health of children and adolescents aged 9–18 across China. In 2019, data were collected through physical examinations and questionnaires from 30 provinces, using stratified cluster sampling. Three regions were selected randomly in each province, followed by random selection of schools and classes for health assessments. The data collection process has also been described in previous studies [19]. This study aggregated data from 229,238 participants. Following data cleaning and quality control procedures, which involved handling missing values, identifying and removing outliers, and excluding cities with fewer than 100 participants, a total of 160,205 subjects from 84 cities across 30 provinces and regions were retained for analysis, as depicted in Figure 1.

### 2.2. Questionnaires, Physical Examination, and Definition of Severe Obesity

Before conducting the physical examination, a comprehensive survey was conducted to gather essential data on each child’s demographics and lifestyle habits. This included inquiries about their gender, age, residency, socioeconomic context, dietary habits (specifically, breakfast and milk consumption), physical activity levels during school hours, screen time, and sleep patterns. Physical examinations were standardized across sites, with trained staff measuring children’s height and weight using calibrated tools. The children’s BMIs were calculated as their body weight (kg) divided by their height (m) squared (kg/m^2^), and their nutritional statuses were categorized according to WHO standards, classifying children into underweight, healthy weight, overweight, obesity, and severe obesity based on sex-specific BMI-for-age percentiles according to the classification method of the Centers for Disease Control and Prevention, in which severe obesity was also divided into class 2 obesity and class 3 obesity [20].

### 2.3. Exposure Assessments

For exposure assessments, air pollution data were sourced from the Tracking Air Pollution in China dataset (http://tapdata.org.cn, accessed on 15 January 2024) [21,22,23,24], which models daily concentrations of PM_2.5_ and five major components (sulfate [SO_4_^2−^], nitrate [NO_3_^−^], ammonium [NH_4_^+^], organic matter [OM], and black carbon [BC]) using observations and satellite data. This database utilizes daily surface PM_2.5_ concentration data at a 10-kilometer resolution in China and derives PM_2.5_ component concentration data from this foundation. Personal exposure refers to the degree to which an individual is exposed to and absorbs pollutants from the environment in their daily lives. The study matched PM_2.5_ and its components’ levels with each child’s examination location and time, calculating annual averages as personal long-term exposure levels. To ensure the continuity of exposure, only children who did not change their place of residence within one year were included in the analysis. Temperature and relative humidity data was sourced from the China Meteorological Data Network (https://data.cma.cn/metadata, accessed on 20 December 2023), and GDP per capita data was sourced from the China Statistical Yearbook [25]. The air pollution data and meteorological data were matched with the health data of children and adolescents based on addresses of schools.

### 2.4. Statistical Analysis 

Differences in baseline characteristics between children of different sexes were assessed using *t*-tests and chi-squared tests. Logistic regression models estimated the odds of overweight and severe obesity associated with per interquartile range (IQR) increase in PM_2.5_ and its components. Additional logistic regression models examined the increased odds of overweight and severe obesity under different quartiles of pollution levels compared to the first quartile. Subgroup analyses were conducted to explore the impact of PM_2.5_ and its components on severe obesity across different factor groups. Models were adjusted for potential confounders, including household registration (urban, rural), socioeconomic status (upper, intermediate, lower, or high school), eating breakfast every day (yes, no), drinking milk every day (yes, no), physical exercise time at school (less than one hour, greater than or equal to one hour), screening time (less than two hours, greater than or equal to two hours), GDP per capita (Chinese yuan) of the city of residence, and annual average temperature (°C). Statistical analyses were conducted using SPSS 24. A *p*-value < 0.05 indicated statistical significance. 

## 3. Results

### 3.1. Children’s Characteristics and Descriptive Statistics of the Exposure and Health Data

Of the 160,205 children included in the analysis, 80,399 were boys (50.2%) and 79,806 were girls (49.8%). The average ages of the boys and girls were 13.5 ± 2.89 years and 13.4 ± 2.86 years, respectively, and the mean BMIs were 20.3 ± 4.05 kg/m^2^ and 19.7 ± 3.55 kg/m^2^, respectively. The numbers of boys and girls with severe obesity were 2928 (3.6%) and 960 (1.2%), respectively. In total, 50.8% of the boys and 50.1% of the girls lived in urban areas. The proportion of boys who ate breakfast and drank milk every day was higher than that of girls, while the proportion of boys who did less than 1 h of physical exercise at school was lower than that of girls. The details can be found in Table 1.

**Figure 2:**
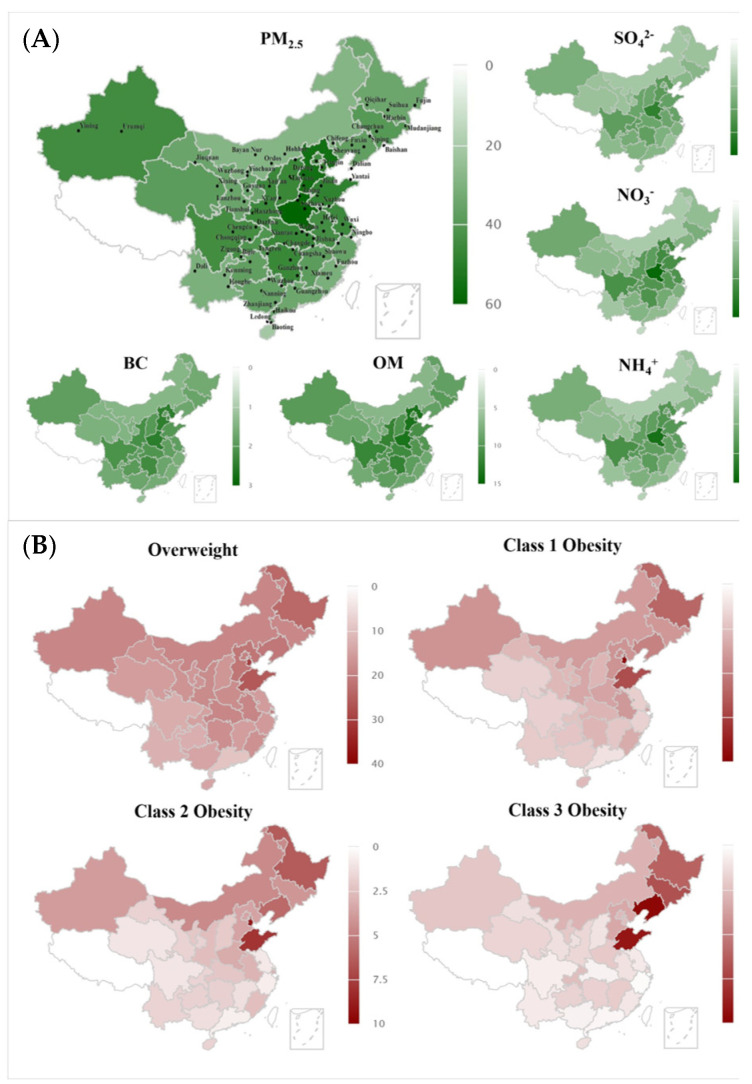
Spatial distribution of study cities and demonstration of average PM_2.5_ exposure of participants. (**A**) Distribution of PM_2.5_ and its components. (**B**) Distribution of obesity at different levels.

The median personal exposure concentrations of PM_2.5_ which included SO_4_^2−^, NO_3_^−^, NH_4_^+^, OM, and BC were 43.0 μg/m^3^, 6.3 μg/m^3^, 8.5 μg/m^3^, 5.3 μg/m^3^, 10.3 μg/m^3^, and 1.9 μg/m^3^, respectively. The IQR personal exposure concentrations of PM_2.5_ which also included SO_4_^2−^, NO_3_^−^, NH_4_^+^, OM, and BC were 11.6 μg/m^3^, 1.7 μg/m^3^, 2.9 μg/m^3^, 1.6 μg/m^3^, 2.9 μg/m^3^, and 0.4 μg/m^3^. The details can be found in Table 2. The spatial distribution of study cities and demonstration of average PM_2.5_ exposure of participants can be found in Figure 2. The distribution of long-term exposure levels of PM_2.5_ and its components in the study cities and correlation between PM_2.5_ and its components can be found in Tables S1 and S2 in Supplementary Materials. 

### 3.2. Effects of Long-Term Exposure to PM_2.5_ and Its Components on Overweight and Obesity

**Figure 3:**
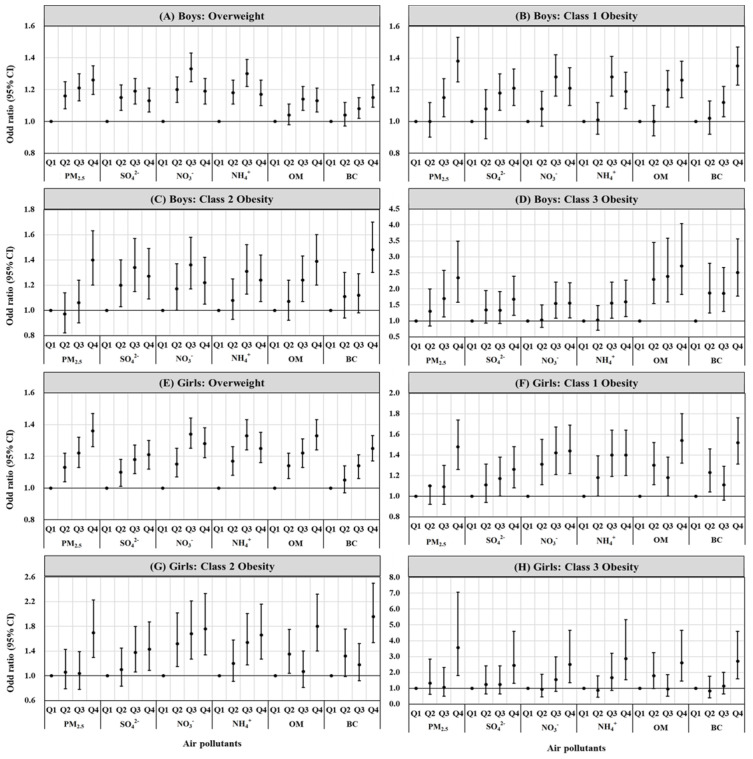
Odd ratios of overweight, class 1 obesity, class 2 obesity, and class 3 obesity in the higher quartile groups compared to the lowest quartile group.

Table 3 shows the increased odds of overweight, class 1 obesity, class 2 obesity, and class 3 obesity per IQR increase in exposure to PM_2.5_ and its components in boys and girls. Overall, the long-term exposure to PM_2.5_ and its components had a more pronounced effect on childhood overweight and obesity in girls. Among the components of PM_2.5_, OM had the most significant impact on overweight and obesity in boys, with an odds ratio (OR) for class 3 obesity per IQR increase in OM concentration at 1.38 (95% CI: 1.20, 1.60) in boys. For girls, NO_3_^−^ was the component most significantly associated with class 1 and class 2 obesity, while NH_4_^+^ was the component most significantly associated with class 3 obesity; the OR for class 3 obesity per IQR increase in NH_4_^+^ concentration was 1.63 (95% CI: 1.31, 2.02) in girls. Figure 3 illustrates the odds of overweight and various classes of obesity in the higher air pollutants quartile groups compared to the lowest quartile group, showing higher odds of overweight and obesity in the higher quartile groups compared to the lowest quartile group in both boys and girls. The specific effect values can be seen in Tables S3 and S4 in Supplementary Materials.

### 3.3. Effects of Long-Term Exposure to PM_2.5_ and Its Components on Severe Obesity in Different Subgroups

**Figure 4:**
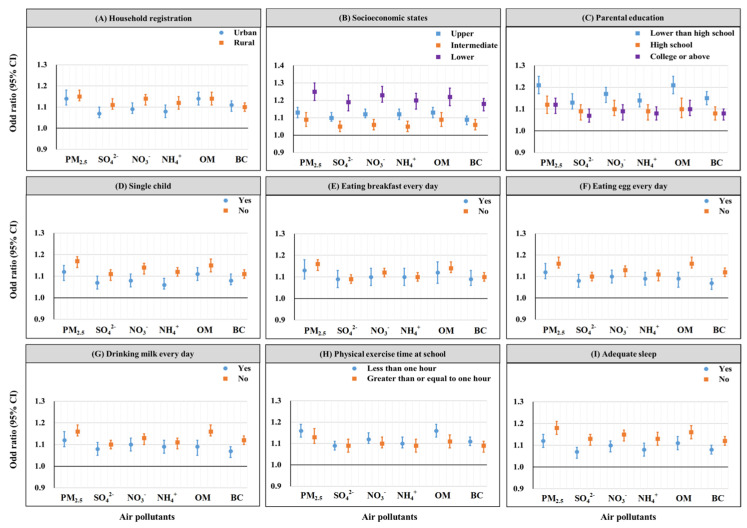
Odd ratios of severe obesity for per IQR increase in exposure to PM_2.5_ and its components in each subgroup.

Figure 4 illustrates the association of PM_2.5_ and its components with the odds of severe obesity in different subgroups. Compared to urban children, the influence of PM_2.5_ including SO_4_^2−^, NO_3_^−^, and NH_4_^+^ on severe obesity was more pronounced in rural children. Conversely, the odds of severe obesity associated with BC were higher in urban children. The odds ratios for severe obesity in children and adolescents per IQR increase in BC exposure were 1.28 (95% CI: 1.11, 1.48) in urban areas and 1.31 (95% CI: 1.16, 1.49) in rural areas. The impact of PM_2.5_ and its components on severe obesity was higher in children from lower economic status households. Lower parental education levels were associated with higher odds of severe obesity in children exposed to PM_2.5_ and its components, with OM being the most influential component. Children who were the single child and those who ate breakfast daily had lower odds of developing severe obesity as a result of long-term exposure to PM_2.5_ and its components. Children with less than 1 h of physical exercise at school and inadequate sleep were at higher odds of severe obesity when exposure to higher PM_2.5_ and its components. The specific effect values can be seen in Table S5 in Supplementary Materials.

## 4. Discussion

This study investigated the potential association between long-term exposure to PM_2.5_ and its components and severe childhood obesity. The results indicate that long-term exposure to PM_2.5_ may increase the odds of severe childhood obesity, with this effect being more pronounced in girls. Among boys, the component with the most significant impact on severe obesity was OM, while NO_3_^−^ and NH_4_^+^ dominated the effect of PM_2.5_ on severe obesity in girls. Furthermore, the impact of PM_2.5_ and its components on severe obesity was greater in children from low economic and low parental education level households. Children with unhealthy dietary and lifestyle habits had higher odds of severe obesity due to long-term exposure to PM_2.5_ and its components. To the best of our knowledge, this is the first nationwide study to explore the association between long-term exposure to PM_2.5_ and its components and severe childhood obesity. The findings highlight the importance of considering the effects of different PM_2.5_ components when addressing severe childhood obesity, emphasizing the need to pay attention to these factors when addressing childhood obesity.

Previous studies have explored the relationship between PM_2.5_ components and overweight/obesity. For example, a longitudinal study in China found that long-term exposure to SO_4_^2−^, NO_3_^−^, NH_4_^+^, OM, and BC may increase the risk of overweight/obesity in children [14], which is similar to the findings of this study. In addition, this study provides additional evidence linking long-term exposure to PM_2.5_ and its components with severe childhood obesity. The results of this study show that long-term exposure to OM increases the odds of severe obesity in boys, while long-term exposure to NO_3_^−^ and NH_4_^+^ increases the odds of severe obesity in girls. The reasons for these differences may involve complex mechanisms such as metabolism and hormonal regulation. For instance, long-term exposure to OM may affect male reproductive health and hormone levels, further altering boys’ energy metabolism and making them more prone to fat accumulation [26]. Currently, there is no evidence of the effects of NO_3_^−^ and NH_4_^+^ on the odds of obesity in girls. However, studies have reported that girls may be more sensitive to air pollution exposure than boys [27], which is consistent with the findings of this study where girls had higher odds of severe obesity due to long-term exposure to PM_2.5_ and its components than boys. These findings provide valuable insights into the specific effects of different PM_2.5_ components on severe childhood obesity and highlight the potential gender-specific vulnerabilities in response to long-term exposure to air pollution.

A survey involving 30 provinces in China has shown that exposure to BC is associated with an increased risk of childhood obesity and overweight, with this effect being more pronounced in urban areas [28], which is consistent with the findings of this study. This association may be related to the fact that BC primarily originates from vehicle exhaust and other traffic emissions [29], exacerbating urban air pollution due to high population density and busy traffic. Children have a faster respiratory rate compared to adults, and their immune and respiratory systems are still developing, making them more sensitive to air pollution [30]. The risks of childhood obesity caused by pollution from traffic emissions need more attention. Furthermore, the impact of several other PM_2.5_ components on severe childhood obesity is more pronounced in rural areas, possibly related to economic levels. Unhealthy lifestyles such as skipping breakfast, lack of physical activity, and insufficient sleep may increase the impact of PM_2.5_ and its components on severe childhood obesity in this study. Skipping breakfast can lead to metabolic disorders, increasing cravings for unhealthy foods and subsequently causing obesity [31]. Additionally, insufficient physical activity reduces energy expenditure, making fat accumulation more likely [32]. Lastly, inadequate sleep can affect hormone levels by increasing ghrelin and reducing leptin, increasing hunger and appetite, potentially leading to weight gain [33]. The combined effects of these factors may make children more vulnerable to the negative impact of PM_2.5_, highlighting the importance of promoting children’s health through measures such as education, healthy diet, and physical activity [34,35].

The main strength of this study lies in its nationwide representativeness, as it is the first to explore the potential association between long-term exposure to PM_2.5_ and its components and severe obesity in children, providing a new perspective and reference for future intervention measures to reduce severe childhood obesity. However, this study also has limitations. For instance, it only focused on several components of PM_2.5_ and their impact on severe childhood obesity, while other pollutants that could potentially influence the results, such as carbon monoxide, were not included in this study. Additionally, as a cross-sectional study, this research cannot establish causal relationships, and the findings need further confirmation through subsequent prospective studies.

## 5. Conclusions

This study found that long-term exposure to PM_2.5_ and its components may be associated with higher odds of severe obesity in children. Children from lower socioeconomic backgrounds and with unhealthy lifestyle habits are more sensitive to long-term exposure to PM_2.5_ and its components. These findings provide evidence for the formulation of policies to improve severe childhood obesity, indicating that healthy lifestyle habits may reduce the odds of severe obesity resulting from exposure to PM_2.5_ and its components. Greater attention should be paid to children living in economically underdeveloped areas, as well as children with unhealthy lifestyle habits, especially those residing in rural areas and those lacking sufficient physical exercise and sleep. Additionally, children’s exposure to traffic-related pollutants should be closely monitored.

## Figures and Tables

**Table 1:** Characteristics of study population.

Characteristics	Boys	Girls	*p* Value
	N = 80,399	N = 79,806	
Age (years) (mean ± SD)	13.5 ± 2.89	13.4 ± 2.86	0.313
BMI (mean ± SD)	20.3 ± 4.05	19.7 ± 3.55	<0.001
Nutritional status (N, %)			<0.001
Normal weight and thinness	56,871 (70.7)	65,611 (82.2)	
Overweight	14,376 (17.9)	11,117 (13.9)	
Class 1 Obesity	6224 (7.7%)	2118 (2.7)	
Class 2 Obesity	2512 (3.1)	809 (1.0)	
Class 3 Obesity	416 (0.5)	151 (0.2)	
Household registration (N, %)			0.013
Urban	40,812 (50.8)	40,018 (50.1)	
Rural	39,587 (49.2)	39,788 (49.9)	
Socioeconomic states (N, %)			0.402
Upper	26,093 (32.5)	25,921 (32.5)	
Intermediate	27,478 (34.2)	27,389 (34.3)	
Lower	25,541 (31.8)	25,299 (31.7)	
High school	1287 (1.6)	1197 (1.5)	
Parental education (N, %)			<0.001
Lower than high school	31,150 (38.7)	31,625 (39.6)	
High school	19,786 (24.6)	19,785 (24.8)	
College or above	24,953 (31.0)	24,432 (30.6)	
Unknown	4510 (5.6)	3964 (5.0)	
Single child (N, %)	36,301 (45.2)	30,393 (38.1)	<0.001
Eating breakfast every day (N, %)	16,090 (20.0)	12,700 (15.9)	<0.001
Eating egg every day (N, %)	27,966 (34.8)	24,802 (31.1)	<0.001
Drinking milk every day (N, %)	27,966 (34.8)	24,802 (31.1)	<0.001
Sugary beverages (N, %)			<0.001
Less than once per day	61,292 (76.2)	64,925 (81.4)	
Greater than or equal to once a day	19,107 (23.8)	14,881 (18.6)	
Physical exercise time at school (N, %)			<0.001
Less than one hour	45,827 (57.0)	48,217 (60.4)	
Greater than or equal to one hour	34,572 (43.0)	31,589 (39.6)	
Adequate sleep (N, %)	37,256 (46.3)	38,653 (48.4)	<0.001
GDP per capita (CNY) (mean ± SD)	81,327 ± 36,427	81,304 ± 34,656	0.894

Abbreviations: BMI, body mass index; CNY, Chinese Yuan; GDP, gross domestic product; SD, standard deviation.

**Table 2:** Description of the long-term exposure of the study participants to air pollutants and meteorological factors.

Exposure Factor	Mean ± SD	5th	25th	50th	75th	95th	IQR
PM_2.5_ (μg/m^3^)	40.2 ± 10.81	23.6	34.5	43.0	46.0	61.1	11.6
SO_4_^2−^ (μg/m^3^)	6.5 ± 1.72	4.2	5.9	6.3	7.6	10.1	1.7
NO_3_^−^ (μg/m^3^)	8.3 ± 3.00	3.6	7.0	8.5	9.9	14.0	2.9
NH_4_^+^ (μg/m^3^)	5.4 ± 1.74	2.7	4.7	5.3	6.4	8.8	1.6
OM (μg/m^3^)	9.8 ± 2.44	6.8	8.3	10.3	11.2	14.7	2.9
BC (μg/m^3^)	1.8 ± 0.42	1.3	1.7	1.9	2.0	2.7	0.4
Temperature (°C)	14.5 ± 4.92	6.3	14.2	15.4	17.1	22.9	2.9
Relative humidity (%)	67.2 ± 10.54	47	59	70	75	82	16

Abbreviations: BC, black carbon; IQR, interquartile range; OM, organic matter; SD, standard deviation.

**Table 3:** Associations for overweight, class 1 obesity, class 2 obesity, and class 3 obesity with per IQR increase in exposure to PM_2.5_ and its components.

Gender	Air Pollutants	Odd Ratio (95% CI)			
		Overweight	Class 1 Obesity	Class 2 Obesity	Class 3 Obesity
Boys	PM_2.5_	1.10 (1.06, 1.13)	1.17 (1.13, 1.22)	1.18 (1.11, 1.26)	1.41 (1.22, 1.64)
	SO_4_^2−^	1.04 (1.02, 1.07)	1.11 (1.07, 1.15)	1.13 (1.07, 1.20)	1.29 (1.13, 1.46)
	NO_3_^−^	1.07 (1.04, 1.10)	1.12 (1.08, 1.17)	1.13 (1.07, 1.20)	1.32 (1.15, 1.50)
	NH_4_^+^	1.06 (1.03, 1.08)	1.11 (1.07, 1.15)	1.12 (1.06, 1.19)	1.28 (1.12, 1.46)
	OM	1.08 (1.05, 1.12)	1.17 (1.12, 1.22)	1.15 (1.08, 1.22)	1.38 (1.20, 1.60)
	BC	1.06 (1.03, 1.08)	1.13 (1.09, 1.16)	1.13 (1.07, 1.18)	1.27 (1.14, 1.42)
Girls	PM_2.5_	1.15 (1.11, 1.18)	1.21 (1.13, 1.29)	1.40 (1.25, 1.56)	1.55 (1.21, 1.99)
	SO_4_^2−^	1.10 (1.06, 1.12)	1.17 (1.10, 1.24)	1.31 (1.19, 1.44)	1.51 (1.23, 1.85)
	NO_3_^−^	1.12 (1.09, 1.15)	1.19 (1.12, 1.26)	1.36 (1.23, 1.50)	1.62 (1.30, 2.03)
	NH_4_^+^	1.10 (1.07, 1.14)	1.17 (1.10, 1.24)	1.32 (1.20, 1.46)	1.63 (1.31, 2.02)
	OM	1.14 (1.10, 1.17)	1.19 (1.11, 1.28)	1.32 (1.18, 1.48)	1.39 (1.08, 1.78)
	BC	1.09 (1.07, 1.12)	1.16 (1.10, 1.22)	1.26 (1.16, 1.37)	1.33 (1.10, 1.60)

Note: Estimates were adjusted for age, household registration, socioeconomic states, parental education, single child, eating breakfast every day, eating egg every day, drinking milk every day, sugary beverages, physical exercise time at school, adequate sleep, GDP per capita, temperature, and relative humidity. Abbreviations: BC, black carbon; GDP, gross domestic product; IQR, interquartile range; OM, organic matter.

## Data Availability

The original contributions presented in the study are included in the article; further inquiries can be directed to the corresponding authors.

## Supplementary Materials

The following supporting information can be downloaded at: https://www.mdpi.com/article/10.3390/children11121536/s1; Table S1: Distribution of long-term exposure levels of PM_2.5_ and its components in the study cities; Table S2: The correlation between PM_2.5_ and its components; Table S3: Adjusted odd ratios of overweight, class 1 obesity, class 2 obesity, and class 3 obesity in the groups with higher quartiles compared with the group with the lowest quartile of pollutants in boys; Table S4: Adjusted odd ratios of overweight, class 1 obesity, class 2 obesity, and class 3 obesity in the groups with higher quartiles compared with the group with the lowest quartile of pollutants in girls; Table S5: Adjusted odd ratios of overweight and obesity for per IQR increase in exposure to PM_2.5_ and its components in each subgroup.
