# Supplementary material for: Association Between Long Term Exposure to PM2.5 and Its Components on Severe Obesity in Chinese Children and Adolescents: A National Study in China
Source: Children (Basel). 2024 Dec 18;11(12):1536. doi: 10.3390/children11121536 (PMC11726891; doi:10.3390/children11121536)
Supplement: Supplementary file 1 [file children-11-01536-s001.zip › children-3308756-supplementary.pdf]

Supplementary materials

Long term effects of $PM_{2.5}$ and its components on severe obesity in Chinese children and adolescents: A National Study in China

Table of Contents

Table S1. Distribution of long-term exposure levels of $PM_{2.5}$ and its components in the study cities.

Table S2. The correlation between $PM_{2.5}$ and its components.

Table S3. Adjusted odd ratios of overweight, class 1 obesity, class 2 obesity, and class 3 obesity in the groups with higher quartiles compared with the group with the lowest quartile of pollutants in boys.

Table S4. Adjusted odd ratios of overweight, class 1 obesity, class 2 obesity, and class 3 obesity in the groups with higher quartiles compared with the group with the lowest quartile of pollutants in girls.

Table S5. Adjusted odd ratios of overweight and obesity for per IQR increase in exposure to PM2.5 and its components in each subgroup.

Table S1. Distribution of long-term exposure levels of $PM_{2.5}$ and its components in the study cities.

City	$PM_{2.5}$ (μg/m^3)			SO_4^{2-} (μg/m^3)			NO_3^- (μg/m^3)			NH_4^+ (μg/m^3)			OM (μg/m^3)			BC (μg/m^3)		
	P_5	P_{50}	P_{95}	P_5	P_{50}	P_{95}	P_5	P_{50}	P_{95}	P_5	P_{50}	P_{95}	P_5	P_{50}	P_{95}	P_5	P_{50}	P_{95}
Baishan	28.3	28.3	28.5	5.1	5.1	5.2	6.9	6.9	7.0	4.6	4.6	4.6	6.3	6.3	6.4	1.3	1.3	1.3
Baoting	14.3	16.8	16.8	3.4	3.9	3.9	2.2	2.5	2.5	2.0	2.2	2.2	4.5	5.3	5.3	1.0	1.2	1.2
Bayan Nur	32.1	33.5	33.5	4.4	4.5	4.5	4.5	4.7	4.7	3.0	3.2	3.2	7.8	8.3	8.3	1.5	1.6	1.6
Beijing	31.5	41.9	56.4	5.2	6.9	8.4	7.1	9.7	12.3	4.5	6.1	7.6	7.2	11.2	12.9	1.4	2.0	2.3
Bijie	28.4	28.4	28.4	6.3	6.3	6.3	5.1	5.1	5.1	3.6	3.6	3.6	8.3	8.3	8.3	1.9	1.9	1.9
Changchun	35.4	38.4	43.3	5.1	5.4	6.2	6.0	7.1	8.7	3.9	4.5	5.3	9.3	10.1	10.6	1.7	1.8	1.9
Changde	43.6	46.4	47.3	7.7	8.2	8.3	10.4	11.1	11.3	6.5	7.0	7.1	11.6	12.3	12.7	2.2	2.3	2.4
Changsha	41.3	53.8	57.3	6.4	9.5	10.5	9.0	11.4	13.5	5.6	6.7	8.2	9.6	14.4	15.6	1.8	2.7	3.0
Chengdu	44.8	45.4	55.2	7.7	8.0	9.3	11.0	11.5	13.2	6.8	7.3	8.0	11.2	11.4	14.4	2.1	2.1	2.7
Chifeng	19.5	20.4	20.4	4.0	4.0	4.0	3.4	3.4	3.4	2.4	2.4	2.5	5.0	5.5	5.5	1.2	1.2	1.2
Chizhou	25.4	34.0	42.2	4.9	6.3	6.6	5.7	8.2	9.2	4.1	5.5	5.8	6.6	8.3	9.6	1.2	1.4	1.8
Chongqing	31.3	42.7	48.1	6.1	8.0	9.2	7.0	9.5	12.0	5.1	6.2	8.2	8.6	10.3	12.0	1.6	1.9	2.2
Dali	20.9	21.9	21.9	4.7	4.7	4.7	3.1	3.1	3.2	2.7	2.7	2.8	6.7	6.7	6.7	1.5	1.5	1.5
Dalian	35.4	38.4	46.8	6.0	6.3	7.8	8.4	8.5	10.3	5.3	5.4	6.5	7.7	8.2	10.1	1.5	1.7	2.1
Datong	31.7	35.6	37.3	5.0	5.2	5.6	6.2	6.7	7.6	4.1	4.4	5.0	7.1	7.6	7.9	1.3	1.6	1.7
Dazhou	37.3	37.4	38.3	7.1	7.1	7.1	8.5	8.6	8.9	6.2	6.3	6.4	10.5	10.6	10.7	1.8	1.8	1.9
Fujin	16.9	16.9	16.9	2.9	2.9	2.9	3.8	3.8	3.8	2.3	2.3	2.3	4.3	4.3	4.3	1.0	1.0	1.0
Fuxin	30.9	31.8	33.3	5.3	5.6	5.6	6.5	6.8	7.0	4.3	4.5	4.6	8.2	8.3	8.9	1.5	1.6	1.6
Fuzhou	20.8	21.1	21.5	4.4	4.4	4.6	3.2	3.3	3.3	2.3	2.3	2.4	6.5	6.5	6.7	1.3	1.3	1.4
Ganzhou	27.6	29.1	29.5	5.3	5.6	5.8	5.5	5.7	5.8	3.8	4.1	4.4	7.4	7.9	8.3	1.4	1.5	1.6
Guangzhou	29.8	30.1	31.2	5.2	6.2	6.6	3.5	4.9	5.1	2.5	3.9	4.0	7.8	8.2	8.6	1.6	1.7	1.8
Guiyang	32.1	36.0	51.0	7.0	7.6	8.0	5.7	6.3	10.9	3.9	4.2	6.8	9.6	10.5	11.7	2.1	2.1	2.4
Guyuan	30.2	30.7	46.4	4.9	5.0	8.6	6.6	6.7	9.9	4.6	4.7	6.3	7.1	7.2	11.1	1.3	1.3	2.1
Haikou	19.1	20.3	24.2	3.8	4.2	4.4	2.8	3.0	4.2	2.5	2.6	3.1	5.9	6.2	6.9	1.2	1.2	1.3
Handan	66.4	66.6	66.6	10.1	10.1	10.2	14.1	14.3	14.3	8.7	8.8	8.8	16.4	16.7	16.8	3.0	3.0	3.1

Hangzhou	35.6	37.4	43.4	6.6	6.7	7.0	8.4	9.0	9.4	5.1	5.5	5.8	7.9	8.2	10.3	1.5	1.6	1.9
Hanzhong	41.2	43.0	43.5	7.4	7.6	7.8	9.6	9.8	10.2	6.8	7.0	7.2	10.9	11.3	11.5	2.0	2.1	2.1
Harbin	33.9	36.3	39.6	4.5	5.0	5.2	5.4	5.7	7.0	3.4	3.7	4.4	9.8	10.1	11.6	1.6	1.8	2.0
Hefei	43.9	46.8	54.2	6.5	8.1	9.8	9.2	11.5	13.9	5.7	7.2	8.6	10.0	11.1	12.2	1.8	2.0	2.3
Hengyang	46.8	47.6	50.8	8.5	8.6	9.2	11.3	11.6	12.2	6.9	7.1	7.5	12.5	12.7	13.5	2.4	2.4	2.6
Hohhot	32.3	35.0	37.3	4.9	5.2	5.3	6.4	6.8	7.1	4.1	4.3	4.5	7.8	8.4	8.8	1.5	1.6	1.7
Honghe	23.8	46.2	50.3	5.5	6.2	6.6	3.6	8.7	9.4	3.2	5.3	5.7	7.3	11.2	12.3	1.6	2.0	2.1
Huangshi	40.6	49.9	49.9	6.2	8.6	8.6	8.7	12.2	12.2	5.4	7.4	7.4	9.6	12.6	12.6	1.8	2.5	2.5
Jinan	59.8	60.2	63.1	10.2	10.4	10.8	14.5	14.8	15.5	9.0	9.2	9.6	12.2	12.3	13.0	2.3	2.3	2.5
Jinhua	25.6	31.4	33.6	5.4	6.1	6.5	5.7	7.2	7.9	4.0	5.0	5.2	6.3	7.3	7.7	1.2	1.4	1.5
Jining	40.4	43.8	46.3	5.9	6.0	6.3	8.3	8.4	9.0	5.0	5.1	5.4	9.3	10.5	11.2	1.7	1.9	2.0
Jinzhong	44.6	45.2	45.4	7.9	8.0	8.0	10.7	10.9	11.0	6.8	6.9	7.0	11.5	11.6	11.7	2.2	2.2	2.2
Jiujiang	34.8	39.4	42.8	6.4	7.2	7.8	8.5	9.9	10.8	5.7	6.5	7.1	8.7	9.6	10.4	1.5	1.7	1.9
Jiuquan	25.6	26.1	26.2	2.4	2.4	2.4	3.2	3.3	3.3	2.3	2.3	2.3	3.7	3.9	4.0	0.7	0.8	0.8
Kunming	24.5	31.4	31.5	5.2	5.4	6.4	3.6	7.4	7.4	3.1	4.8	4.8	6.9	7.1	8.2	1.3	1.3	1.9
Lanzhou	29.0	36.6	39.5	4.2	5.4	5.9	5.5	6.4	8.3	3.8	4.2	5.2	7.1	8.4	9.7	1.3	1.6	1.8
Ledong	14.1	14.1	16.1	3.3	3.3	3.8	2.2	2.2	2.4	2.0	2.0	2.2	4.5	4.5	5.2	1.0	1.0	1.1
Liuzhou	32.6	32.7	34.1	6.2	6.3	6.3	6.7	6.7	6.8	5.0	5.2	5.2	8.9	9.2	9.2	1.7	1.7	1.7
Mudanjiang	30.3	30.3	30.3	4.5	4.5	4.5	6.2	6.2	6.2	3.8	3.8	3.8	8.0	8.0	8.0	1.5	1.5	1.5
Nanchang	32.9	39.2	44.5	6.1	6.9	8.0	7.8	8.9	10.2	5.4	5.8	6.7	8.4	9.4	10.6	1.5	1.7	2.0
Nanjing	40.8	45.1	46.0	7.4	8.2	8.7	10.3	11.2	11.7	6.5	6.9	7.2	8.6	9.4	9.6	1.6	1.8	1.8
Nanning	31.0	32.3	45.9	5.8	6.0	6.4	5.8	5.9	9.0	4.3	4.4	5.4	8.3	8.4	11.8	1.6	1.6	2.1
Ningbo	26.2	28.4	28.5	5.3	5.7	5.8	5.6	6.0	6.1	3.7	4.0	4.1	6.4	7.0	7.2	1.3	1.4	1.5
Ordos	26.0	28.1	34.7	4.0	4.4	4.8	4.7	5.2	6.4	3.3	3.4	4.1	5.4	5.6	8.1	1.1	1.2	1.6
Qiqihar	22.7	23.2	23.6	3.5	3.5	3.7	3.5	3.6	4.1	2.4	2.4	2.6	6.8	7.0	7.4	1.2	1.3	1.4
Shanghai	27.8	35.2	37.9	5.5	6.9	7.4	6.0	6.9	7.7	4.1	4.6	5.2	6.3	7.6	8.2	1.3	1.7	1.8
Shaoguan	23.3	24.2	28.5	4.7	4.9	5.5	4.7	4.8	5.8	3.6	3.8	4.5	6.7	6.9	7.7	1.3	1.3	1.4
Shaowu	16.0	19.2	21.5	3.5	4.1	4.6	3.0	3.9	4.4	2.4	3.0	3.4	4.6	5.3	5.9	0.9	1.0	1.1
Shenyang	30.7	42.1	42.6	5.1	6.1	6.1	6.4	7.0	7.0	4.1	4.5	4.6	7.9	10.6	10.9	1.4	2.1	2.1

Shijiazhuang	33.8	64.6	72.4	5.5	9.7	10.6	7.7	14.0	15.0	4.9	8.6	9.2	7.7	16.2	19.0	1.5	2.9	3.4
Siping	34.7	37.4	43.7	5.9	6.2	6.2	7.7	8.4	8.5	5.0	5.2	5.4	8.9	9.4	11.0	1.6	1.7	2.0
Suihua	35.1	43.8	46.0	4.8	6.2	6.4	5.9	8.8	9.1	3.7	5.4	5.5	10.6	10.8	11.2	1.9	1.9	2.0
Suqian	42.7	46.1	46.8	8.2	8.8	8.9	11.6	12.5	12.7	7.2	7.7	7.8	9.2	10.0	10.3	1.7	1.9	1.9
Suzhou	44.6	49.0	50.1	8.6	9.0	9.2	12.2	13.2	13.6	7.5	8.2	8.4	9.8	10.7	11.0	1.8	2.1	2.2
Taiyuan	40.1	52.8	54.7	6.2	7.4	8.1	8.5	8.9	11.0	5.3	5.7	7.2	8.3	10.7	11.9	1.6	2.1	2.2
Tangshan	47.6	51.8	57.2	6.7	7.2	8.0	8.5	9.1	10.3	5.4	5.7	6.5	10.9	11.7	13.0	2.0	2.2	2.4
Tianjin	49.1	51.1	52.5	6.8	7.1	7.6	9.6	10.4	10.4	5.8	6.3	6.5	11.5	11.9	12.4	2.1	2.2	2.2
Tianshui	30.6	34.3	48.5	5.4	5.7	9.2	6.9	7.5	11.9	4.9	5.1	8.2	7.9	9.0	11.7	1.4	1.6	2.1
Tongren	31.4	31.4	33.6	6.3	6.3	6.8	6.6	6.6	7.2	4.5	4.5	4.8	9.1	9.1	9.6	1.8	1.8	1.9
Urumqi	49.6	49.8	59.0	7.0	7.1	8.8	9.1	9.1	11.1	5.7	5.7	7.0	9.2	9.2	10.8	1.9	1.9	2.3
Wuhan	41.6	44.0	49.5	5.8	6.1	8.0	8.3	8.6	10.9	5.0	5.2	6.4	10.2	11.0	12.8	1.8	2.0	2.4
Wuxi	35.7	42.8	44.0	6.5	7.7	7.9	8.2	10.1	10.4	5.0	6.2	6.3	7.6	9.2	9.4	1.5	1.8	1.9
Wuzhong	31.4	34.3	35.3	4.6	5.1	5.2	5.5	6.3	6.6	3.7	4.3	4.4	6.7	7.4	7.6	1.4	1.5	1.5
Wuzhou	27.6	27.7	28.5	5.3	5.3	5.4	5.2	5.3	5.4	4.0	4.1	4.2	7.5	7.5	7.7	1.5	1.5	1.5
Xiamen	24.6	24.6	26.7	4.8	4.8	5.1	3.7	3.7	4.1	2.7	2.7	2.7	8.4	8.4	9.2	1.6	1.6	1.8
Xi'an	42.5	44.8	59.4	6.1	6.4	9.5	8.7	9.0	12.6	5.3	5.5	7.7	10.0	10.8	16.8	1.8	1.9	3.1
Xiantao	53.5	53.5	53.7	9.7	9.7	9.8	14.1	14.1	14.2	8.6	8.6	8.6	13.1	13.1	13.2	2.5	2.5	2.5
Xining	33.6	34.0	51.4	3.9	4.1	7.4	5.2	5.6	10.8	3.5	3.8	6.6	7.1	7.3	11.8	1.2	1.3	2.1
Xinxiang	64.5	64.6	65.2	11.3	11.3	11.6	16.1	16.1	16.4	10.1	10.1	10.4	14.2	14.3	14.3	2.7	2.7	2.7
Xuzhou	55.5	55.5	56.9	9.7	9.7	9.8	14.4	14.4	14.6	8.9	8.9	9.1	11.7	11.7	11.9	2.3	2.3	2.3
Yan'an	32.4	32.5	33.6	5.6	5.6	5.8	7.1	7.1	7.3	5.0	5.0	5.2	7.9	8.0	8.1	1.4	1.4	1.4
Yantai	34.4	34.4	38.7	6.3	6.3	6.8	8.3	8.3	9.1	5.4	5.4	5.9	7.5	7.5	8.1	1.5	1.5	1.6
Yinchuan	35.9	42.4	44.6	5.1	5.8	6.0	6.2	8.1	8.4	4.1	4.8	5.0	7.5	10.2	10.9	1.5	1.8	1.9
Yining	29.9	41.0	46.9	4.8	6.3	7.1	5.7	8.9	9.9	3.5	5.4	6.3	7.9	11.5	13.0	1.5	2.0	2.2
Yuncheng	47.8	57.2	57.5	8.3	9.8	10.0	11.3	13.5	13.7	7.3	8.7	8.9	11.6	13.8	13.9	2.1	2.5	2.5
Zhanjiang	23.8	41.7	43.6	4.7	6.3	6.5	3.9	8.9	9.1	3.1	5.5	5.6	7.0	9.8	10.3	1.4	1.8	1.9
Zhengzhou	40.8	51.6	69.0	6.4	8.2	11.9	9.0	11.7	17.1	5.7	7.3	10.6	9.4	11.7	15.3	1.7	2.1	2.9
Zhoukou	61.0	61.6	62.3	11.4	11.5	11.7	16.7	16.8	17.1	10.3	10.3	10.5	14.1	14.2	14.4	2.5	2.6	2.6

Zigong	39.8	45.7	46.4	7.0	8.1	8.4	10.0	11.5	11.7	6.7	7.7	7.9	10.6	12.0	12.2	1.8	2.2	2.2

Abbreviations: BC, black carbon; OM, organic matter.

Table S2. The correlation between $PM_{2.5}$ and its components.

	$PM_{2.5}$	SO_4^{2-}	NO_3^-	NH_4^+	OM	BC
$PM_{2.5}$	1					
SO_4^{2-}	**0.856**	1				
NO_3^-	**0.935**	**0.933**	1			
NH_4^+	**0.892**	**0.954**	**0.985**	1		
OM	**0.926**	**0.830**	**0.859**	**0.829**	1	
BC	**0.903**	**0.879**	**0.846**	**0.825**	**0.970**	1

Note: Bold values referred to $P<0.05$.

Abbreviations: BC, black carbon; OM, organic matter.

Table S3. Adjusted odd ratios of overweight, class 1 obesity, class 2 obesity, and class 3 obesity in the groups with higher quartiles compared with the group with the lowest quartile of pollutants in boys.

Air pollutants	Quartile groups	Odd ratio (95% CI)			
		Overweight	Class 1 Obesity	Class 2 Obesity	Class 3 Obesity
$PM_{2.5}$ (μg/m³)	Q1 (<32.4 μg/m³)	1.00 (reference)	1.00 (reference)	1.00 (reference)	1.00 (reference)
	Q2 (32.4-42.1 μg/m³)	**1.16 (1.08, 1.25)**	1.00 (0.90, 1.12)	0.97 (0.82, 1.14)	1.30 (0.84, 2.00)
	Q3 (42.2-45.7 μg/m³)	**1.21 (1.13, 1.30)**	**1.15 (1.03, 1.27)**	1.06 (0.90, 1.24)	**1.70 (1.12, 2.58)**
	Q4 (>45.7 μg/m³)	**1.26 (1.17, 1.35)**	**1.38 (1.25, 1.53)**	**1.40 (1.20, 1.63)**	**2.35 (1.58, 3.49)**
SO_4^{2-} (μg/m³)	Q1 (<5.5 μg/m³)	1.00 (reference)	1.00 (reference)	1.00 (reference)	1.00 (reference)
	Q2 (5.5-6.2 μg/m³)	**1.15 (1.07, 1.23)**	1.08 (0.89, 1.20)	**1.20 (1.03, 1.40)**	1.34 (0.93, 1.95)
	Q3 (6.3-7.4 μg/m³)	**1.19 (1.11, 1.27)**	**1.18 (1.07, 1.30)**	**1.34 (1.15, 1.57)**	1.33 (0.92, 1.92)
	Q4 (>7.4 μg/m³)	**1.13 (1.06, 1.21)**	**1.21 (1.10, 1.33)**	**1.27 (1.09, 1.49)**	**1.68 (1.17, 2.39)**
NO_3^- (μg/m³)	Q1 (<6.5 μg/m³)	1.00 (reference)	1.00 (reference)	1.00 (reference)	1.00 (reference)
	Q2 (6.5-8.4 μg/m³)	**1.20 (1.12, 1.28)**	1.08 (0.97, 1.19)	**1.17 (1.00, 1.37)**	1.03 (0.80, 1.50)
	Q3 (8.5-9.8 μg/m³)	**1.33 (1.25, 1.43)**	**1.28 (1.16, 1.42)**	**1.36 (1.17, 1.58)**	**1.54 (1.08, 2.21)**
	Q4 (>9.8 μg/m³)	**1.19 (1.11, 1.27)**	**1.21 (1.10, 1.34)**	**1.22 (1.05, 1.42)**	**1.55 (1.09, 2.19)**
NH_4^+ (μg/m³)	Q1 (<4.4 μg/m³)	1.00 (reference)	1.00 (reference)	1.00 (reference)	1.00 (reference)
	Q2 (4.4-5.2 μg/m³)	**1.18 (1.11, 1.26)**	1.01 (0.92, 1.12)	1.08 (0.93, 1.25)	1.03 (0.71, 1.48)
	Q3 (5.3-6.3 μg/m³)	**1.30 (1.22, 1.39)**	**1.28 (1.16, 1.41)**	**1.31 (1.13, 1.52)**	**1.55 (1.08, 2.21)**
	Q4 (>6.3 μg/m³)	**1.17 (1.10, 1.26)**	**1.19 (1.08, 1.31)**	**1.24 (1.07, 1.44)**	**1.60 (1.13, 2.27)**
OM (μg/m³)	Q1 (<7.9 μg/m³)	1.00 (reference)	1.00 (reference)	1.00 (reference)	1.00 (reference)
	Q2 (7.9-10.1 μg/m³)	1.04 (0.98, 1.11)	1.00 (0.91, 1.10)	1.07 (0.92, 1.24)	**2.30 (1.54, 3.45)**
	Q3 (10.2-11.1 μg/m³)	**1.14 (1.07, 1.22)**	**1.20 (1.09, 1.32)**	**1.24 (1.07, 1.43)**	**2.39 (1.59, 3.58)**
	Q4 (>11.1 μg/m³)	**1.13 (1.06, 1.21)**	**1.26 (1.15, 1.38)**	**1.39 (1.20, 1.60)**	**2.71 (1.82, 4.04)**
BC (μg/m³)	Q1 (<1.6 μg/m³)	1.00 (reference)	1.00 (reference)	1.00 (reference)	1.00 (reference)

Q2 (1.6-1.8 μg/m³)	1.04 (0.97, 1.12)	1.02 (0.92, 1.13)	1.11 (0.94, 1.30)	**1.87 (1.25, 2.80)**
Q3 (1.9-2.0 μg/m³)	**1.08 (1.02, 1.15)**	**1.12 (1.03, 1.22)**	1.12 (0.98, 1.29)	**1.86 (1.30, 2.67)**
Q4 (>2.0 μg/m³)	**1.15 (1.09, 1.23)**	**1.35 (1.23, 1.47)**	**1.48 (1.30, 1.70)**	**2.51 (1.77, 3.56)**

Note: Estimates were adjusted for age, household registration, socioeconomic states, parental education, single child, eating breakfast every day, eating egg every day, drinking milk every day, sugary beverages, physical exercise time at school, adequate sleep, GDP per capita, temperature and relative humidity. Bold values referred to P<0.05.

Abbreviations: BC, black carbon; GDP, gross domestic product; OM, organic matter.

Table S4. Adjusted odd ratios of overweight, class 1 obesity, class 2 obesity, and class 3 obesity in the groups with higher quartiles compared with the group with the lowest quartile of pollutants in girls.

Air pollutants	Quartile groups	Odd ratio (95% CI)			
		Overweight	Class 1 Obesity	Class 2 Obesity	Class 3 Obesity
$PM_{2.5}$ (μg/m³)	Q1 (<32.4 μg/m³)	1.00 (reference)	1.00 (reference)	1.00 (reference)	1.00 (reference)
	Q2 (32.4-42.1 μg/m³)	**1.13 (1.04, 1.22)**	1.10 (0.92, 1,31)	1.06 (0.79, 1.43)	1.32 (0.61, 2.83)
	Q3 (42.2-45.7 μg/m³)	**1.22 (1.13, 1.32)**	1.09 (0.92, 1.30)	1.04 (0.78, 1.39)	1.06 (0.49, 2.31)
	Q4 (>45.7 μg/m³)	**1.36 (1.26, 1.47)**	**1.48 (1.26, 1.74)**	**1.70 (1.30. 2.23)**	**3.56 (1.80, 7.05)**
SO_4^{2-} (μg/m³)	Q1 (<5.5 μg/m³)	1.00 (reference)	1.00 (reference)	1.00 (reference)	1.00 (reference)
	Q2 (5.5-6.2 μg/m³)	**1.10 (1.01, 1.18)**	1.11 (0.94, 1.31)	1.10 (0.83, 1.45)	1.23 (0.63, 2.42)
	Q3 (6.3-7.4 μg/m³)	**1.18 (1.09, 1.27)**	1.17 (1.00, 1.38)	**1.38 (1.06, 1.80)**	1.25 (0.64, 2.42)
	Q4 (>7.4 μg/m³)	**1.21 (1.12, 1.30)**	**1.26 (1.08, 1.48)**	**1.43 (1.09, 1.87)**	**2.45 (1.31, 4.59)**
NO_3^- (μg/m³)	Q1 (<6.5 μg/m³)	1.00 (reference)	1.00 (reference)	1.00 (reference)	1.00 (reference)
	Q2 (6.5-8.4 μg/m³)	**1.15 (1.07, 1.25)**	**1.31 (1.11, 1.55)**	**1.52 (1.15, 2.02)**	0.93 (0.46, 1.88)
	Q3 (8.5-9.8 μg/m³)	**1.34 (1.25, 1.44)**	**1.42 (1.21, 1.67)**	**1.68 (1.27, 2.21)**	1.55 (0.81, 2.98)
	Q4 (>9.8 μg/m³)	**1.28 (1.19, 1.38)**	**1.44 (1.22, 1.69)**	**1.76 (1.34, 2.33)**	**2.51 (1.35, 4.66)**
NH_4^+ (μg/m³)	Q1 (<4.4 μg/m³)	1.00 (reference)	1.00 (reference)	1.00 (reference)	1.00 (reference)
	Q2 (4.4-5.2 μg/m³)	**1.17 (1.08, 1.26)**	**1.18 (1.00, 1.39)**	1.20 (0.91, 1.58)	0.87 (0.43, 1.79)
	Q3 (5.3-6.3 μg/m³)	**1.33 (1.24, 1.43)**	**1.40 (1.19, 1.64)**	**1.54 (1.18, 2.01)**	1.67 (0.87, 3.20)
	Q4 (>6.3 μg/m³)	**1.25 (1.16, 1.35)**	**1.40 (1.20, 1.64)**	**1.66 (1.27, 2.16)**	**2.86 (1.53, 5.33)**
OM (μg/m³)	Q1 (<7.9 μg/m³)	1.00 (reference)	1.00 (reference)	1.00 (reference)	1.00 (reference)
	Q2 (7.9-10.1 μg/m³)	**1.14 (1.06, 1.22)**	**1.30 (1.11, 1.52)**	**1.35 (1.04, 1.75)**	1.78 (0.98, 3.24)

	Q3 (10.2-11.1 μg/m³)	**1.22 (1.13, 1.31)**	1.18 (1.00, 1.38)	1.07 (0.81, 1.40)	0.95 (0.49. 1.87)
	Q4 (>11.1 μg/m³)	**1.33 (1.24, 1.43)**	**1.54 (1.32, 1.80)**	**1.80 (1.40, 2.32)**	**2.60 (1.46, 4.65)**
BC (μg/m³)	Q1 (<1.6 μg/m³)	1.00 (reference)	1.00 (reference)	1.00 (reference)	1.00 (reference)
	Q2 (1.6-1.8 μg/m³)	1.05 (0.97, 1.14)	**1.23 (1.04, 1.46)**	1.32 (0.99, 1.76)	0.83 (0.39, 1.75)
	Q3 (1.9-2.0 μg/m³)	**1.14 (1.06, 1.21)**	1.11 (0.96, 1.29)	1.18 (0.92, 1.52)	1.14 (0.64, 2.01)
	Q4 (>2.0 μg/m³)	**1.25 (1.17, 1.33)**	**1.52 (1.31, 1.76)**	**1.96 (1.54, 2.50)**	**2.71 (1.59, 4.60)**

Note: Estimates were adjusted for age, household registration, socioeconomic states, parental education, single child, eating breakfast every day, eating egg every day, drinking milk every day, sugary beverages, physical exercise time at school, adequate sleep, GDP per capita, temperature and relative humidity. Bold values referred to P<0.05.

Abbreviations: BC, black carbon; GDP, gross domestic product; OM, organic matter.

Table S5. Adjusted odd ratios of severe obesity for per IQR increase in exposure to PM2.5 and its components in each subgroup.

Subgroup		Odd ratio (95% CI)					
		$PM_{2.5}$	SO_4^{2-}	NO_3^-	NH_4^+	OM	BC
Household registration	Urban	1.14 (1.11, 1.18)	1.07 (1.05, 1.10)	1.09 (1.07, 1.12)	1.08 (1.05, 1.11)	1.14 (1.11, 1.17)	1.11 (1.08, 1.13)
	Rural	1.15 (1.13, 1.18)	1.11 (1.09, 1.14)	1.14 (1.11, 1.16)	1.12 (1.09, 1.15)	1.14 (1.11, 1.17)	1.10 (1.08, 1.12)
Socioeconomic states	Upper	1.13 (1.10, 1.16)	1.10 (1.08, 1.13)	1.12 (1.10, 1.15)	1.12 (1.09, 1.15)	1.13 (1.10, 1.16)	1.09 (1.06, 1.11)
	Intermediate	1.09 (1.05, 1.13)	1.05 (1.02, 1.08)	1.06 (1.03, 1.09)	1.05 (1.02, 1.08)	1.09 (1.05, 1.13)	1.06 (1.03, 1.09)
	Lower	1.25 (1.20, 1.30)	1.19 (1.14, 1.23)	1.23 (1.19, 1.28)	1.20 (1.15, 1.24)	1.22 (1.17, 1.27)	1.18 (1.14, 1.21)
Parental education	Lower than high school	1.21 (1.17, 1.25)	1.13 (1.10, 1.17)	1.17 (1.13, 1.20)	1.14 (1.11, 1.17)	1.21 (1.17, 1.25)	1.15 (1.12, 1.18)
	High school	1.12 (1.08, 1.16)	1.09 (1.05, 1.12)	1.10 (1.07, 1.14)	1.09 (1.05, 1.12)	1.10 (1.06, 1.15)	1.08 (1.05, 1.11)
	College or above	1.12 (1.08, 1.15)	1.07 (1.04, 1.10)	1.09 (1.05, 1.12)	1.08 (1.05, 1.11)	1.10 (1.07, 1.14)	1.08 (1.05, 1.10)
Single child	Yes	1.12 (1.08, 1.15)	1.07 (1.04, 1.10)	1.08 (1.05, 1.11)	1.06 (1.04, 1.09)	1.11 (1.08, 1.14)	1.08 (1.06, 1.11)
	No	1.17 (1.14, 1.19)	1.11 (1.08, 1.13)	1.14 (1.11, 1.16)	1.12 (1.10, 1.14)	1.15 (1.12, 1.18)	1.11 (1.09, 1.13)
Eating breakfast every day	Yes	1.13 (1.09, 1.18)	1.09 (1.05, 1.13)	1.10 (1.06, 1.14)	1.10 (1.06, 1.14)	1.12 (1.07, 1.17)	1.09 (1.06, 1.13)

	No	1.16 (1.13, 1.18)	1.09 (1.07, 1.11)	1.12 (1.10, 1.14)	1.10 (1.08, 1.12)	1.14 (1.12, 1.17)	1.10 (1.08, 1.12)
Eating egg every day	Yes	1.12 (1.09, 1.16)	1.08 (1.05, 1.11)	1.10 (1.07, 1.13)	1.09 (1.06, 1.12)	1.09 (1.05, 1.12)	1.07 (1.04, 1.09)
	No	1.16 (1.14, 1.19)	1.10 (1.08, 1.12)	1.13 (1.10, 1.15)	1.11 (1.08, 1.13)	1.16 (1.14, 1.19)	1.12 (1.10, 1.14)
Drinking milk every day	Yes	1.12 (1.09, 1.16)	1.08 (1.05, 1.11)	1.10 (1.07, 1.13)	1.09 (1.06, 1.12)	1.09 (1.05, 1.12)	1.07 (1.04, 1.09)
	No	1.16 (1.14, 1.19)	1.10 (1.08, 1.12)	1.13 (1.10, 1.15)	1.11 (1.08, 1.13)	1.16 (1.14, 1.19)	1.12 (1.10, 1.14)
Sugary beverages	Less than once per day	1.17 (1.14, 1.19)	1.10 (1.08, 1.12)	1.13 (1.11, 1.15)	1.11 (1.09, 1.13)	1.15 (1.13, 1.18)	1.11 (1.09, 1.13)
	Greater than or equal to once a day	1.09 (1.04, 1.13)	1.05 (1.01, 1.09)	1.07 (1.03, 1.11)	1.06 (1.02, 1.09)	1.08 (1.03, 1.12)	1.05 (1.02, 1.09)
Physical exercise time at school	Less than one hour	1.16 (1.13, 1.19)	1.09 (1.07, 1.11)	1.12 (1.10, 1.15)	1.10 (1.08, 1.13)	1.16 (1.13, 1.19)	1.11 (1.09, 1.13)
	Greater than or equal to one hour	1.13 (1.10, 1.17)	1.09 (1.06, 1.12)	1.10 (1.08, 1.13)	1.09 (1.06, 1.12)	1.11 (1.08, 1.14)	1.09 (1.06, 1.11)
Adequate sleep	Yes	1.12 (1.09, 1.15)	1.07, 1.04, 1.09)	1.10 (1.07, 1.12)	1.08 (1.05, 1.11)	1.11 (1.08, 1.14)	1.08 (1.06, 1.10)
	No	1.18 (1.15, 1.21)	1.13 (1.10, 1.15)	1.15 (1.12, 1.17)	1.13 (1.10, 1.16)	1.16 (1.13, 1.19)	1.12 (1.10, 1.14)

Note: Estimates were adjusted for age, gender, household registration, socioeconomic states, parental education, single child, eating breakfast every day, eating egg every day, drinking milk every day, sugary beverages, physical exercise time at school, adequate sleep, GDP per capita, temperature and relative humidity. Bold values referred to P<0.05.

Abbreviations: BC, black carbon; GDP, gross domestic product; IQR, interquartile range; OM, organic matter.
